# Complex genome evolution in *Anopheles coluzzii* associated with increased insecticide usage in Mali

**DOI:** 10.1111/mec.13382

**Published:** 2015-10-06

**Authors:** Bradley J. Main, Yoosook Lee, Travis C. Collier, Laura C. Norris, Katherine Brisco, Abdrahamane Fofana, Anthony J. Cornel, Gregory C. Lanzaro

**Affiliations:** ^1^ Vector Genetics LaboratoryUC Davis1089 Veterinary Medicine Dr, 4225 VM3BDavisCA95616USA; ^2^ Department of Pathology, Microbiology and ImmunologyUC Davis1089 Veterinary Medicine Dr, 4225 VM3BDavisCA95616USA; ^3^ Department of Entomology and NematologyUniversity of CaliforniaDavisCA95616USA; ^4^ Malaria Research and Training CenterUniversity of BamakoBP 1805BamakoMali

**Keywords:** adaptive introgression, *Anopheles*, epistasis, hybridization, insecticide resistance, *kdr*, malaria vector, P450

## Abstract

In certain cases, a species may have access to important genetic variation present in a related species via adaptive introgression. These novel alleles may interact with their new genetic background, resulting in unexpected phenotypes. In this study, we describe a selective sweep on standing variation on the X chromosome in the mosquito *Anopheles coluzzii*, a principal malaria vector in West Africa. This event may have been influenced by the recent adaptive introgression of the insecticide resistance gene known as *kdr* from the sister species *Anopheles gambiae*. Individuals carrying both *kdr* and a nearly fixed X‐linked haplotype, encompassing at least four genes including the P450 gene *CYP9K1* and the cuticular protein *CPR125*, have rapidly increased in relative frequency. In parallel, a reproductively isolated insecticide‐susceptible *A. gambiae* population (Bamako form) has been driven to local extinction, likely due to strong selection from increased insecticide‐treated bed net usage.

## Introduction

Leaky interspecies reproductive barriers may increase genetic variability upon which selection can act, increasing the evolutionary responsiveness of a species (Stelkens *et al*. 2014). Adaptive introgression is thought to be most common in plants (Hedrick 2013), but clear examples are emerging in animals. Examples include warfarin resistance in mice (Song *et al*. 2011), coat colour in wolves (Anderson *et al*. 2009), wing coloration patterns in butterflies (Dasmahapatra *et al*. 2012; Pardo‐Diaz *et al*. 2012) and, more recently, insecticide resistance in African malaria mosquitoes (Clarkson *et al*. 2014; Norris *et al*. 2015).

*Anopheles coluzzii* is a newly described species (Coetzee *et al*. 2013) that is morphologically identical to *Anopheles gambiae* (previously referred to as the M and S forms of *A. gambiae,* respectively). Both are major vectors of human malaria and are sympatric throughout much of West and Central Africa (Della Torre *et al*. 2005; Diabaté *et al*. 2009). Divergence is thought to exist at least in part due to adaptation to distinct larval habitats (Gimonneau *et al*. 2012; Kamdem *et al*. 2012). The taxon *A. gambiae* includes two chromosomal forms, known as the Savanna and Bamako form. The two are distinguishable with respect to paracentric chromosome inversion karyotypes, are sympatric in Mali along the Niger and Senegal Rivers and are to a large extent reproductively isolated (Coluzzi *et al*. 1979; Toure *et al*. 1998; Manoukis *et al*. 2008). We will refer to the Savanna form as *A. gambiae* and use the designation Ag‐Bamako for the Bamako form.

Comparisons between *A. coluzzii* and *A. gambiae* genomes have revealed pronounced differentiation at pericentromeric regions on each chromosome (Turner *et al*. 2005; Lawniczak *et al*. 2010; White *et al*. 2010; Reidenbach *et al*. 2012). This result is consistent with a model of speciation that is not strictly dependent on allopatry, namely the ‘speciation islands’ model (Turner *et al*. 2005). Under this model, strong selection on genes important for reproductive isolation maintains divergence at discrete regions, while the remainder of the genome is homogenized by gene flow between sympatric populations (Turner *et al*. 2005; Via & West 2008; Nosil *et al*. 2009; Weetman *et al*. 2012). An alternative hypothesis poses that reduced diversity due to selection on genes within these low recombining regions may have facilitated the fixation of alternative ancestral haplotypes in these regions, resulting in ‘incidental islands’ (Lawniczak *et al*. 2010; Turner & Hahn 2010; White *et al*. 2010). The ‘incidental islands’ hypothesis argues against variable rates of gene flow as the major architect of the islands of divergence. In 2006, the insecticide resistance gene *kdr* and the entire 2L island were stably introgressed from *A. gambiae* into *A. coluzzii* in Selinkenyi, Mali (Clarkson *et al*. 2014; Norris *et al*. 2015). Interestingly, reproductive isolation was quickly re‐established, based on markers on X and 3L (Lee *et al*. 2013b).

Hybrids between *A. coluzzii* and *A. gambiae* are detected in punctuated bursts in Mali, and early‐stage hybrids are typically short lived, presumably due to reduced fitness (Lee *et al*. 2013b). However, cases where hybrids overcame this apparent ‘fitness bottleneck’ in nature and backcrossed with one parental strain (Uecker *et al*. 2014) have been reported in Guinea‐Bissau (Marsden *et al*. 2011), Ghana (Clarkson *et al*. 2014) and Mali (Norris *et al*. 2015). In Mali, a dramatic increase in insecticide‐treated bed net (ITN) usage starting in 2005 (Milliner 2009) likely altered the fitness landscape and promoted adaptive introgression of *kdr* from *A. gambiae* into *A. coluzzii* (Tripet *et al*. 2006; Norris *et al*. 2015). *Kdr* refers to nonsynonymous mutations in the voltage‐gated sodium channel gene (*para*); the most common *kdr* mutation in West Africa is L1014F (Ranson *et al*. 2011). The L1014F mutation confers resistance by altering the binding site of pyrethroid insecticides, a mechanism called target‐site resistance. *Kdr* has been increasing in geographical distribution and relative frequency throughout Africa, apparently in response to increased ITN use (Ranson *et al*. 2009, 2011; Trape *et al*. 2011). Genetic signatures of selection for this introgression (Clarkson *et al*. 2014; Norris *et al*. 2015) and evidence showing that *A. coluzzii* individuals with the introgressed *kdr* (*kdr A. coluzzii*) have increased in relative frequency since 2006 (Norris *et al*. 2015) suggest that this introgression is highly adaptive.

In addition to target‐site resistance, the combination of reduced cuticle penetrance (Ahmad *et al*. 2006; Puinean *et al*. 2010; Wood *et al*. 2010; Willis 2014) and increased activity of metabolic detoxification enzymes like cytochrome P450 genes and glutathione S‐transferases (GSTs) can also confer resistance to insecticides (Hemingway 2000; Hemingway & Ranson 2000; Müller *et al*. 2008; Stevenson *et al*. 2011). For example, a positive association between cuticle thickness and pyrethroid resistance was reported in the closely related mosquito species *A. funestus* (Wood *et al*. 2010). But, most of the identified insecticide resistance genes in *A. gambiae* (119 in all) are P450 genes (64%; Srivastava *et al*. 2010). Gene expression studies in anopheline mosquitoes have reported associations between over expression of several P450 genes and insecticide resistance, including *CYP9K1* (Tene *et al*. 2013; Mulamba *et al*. 2014), *CYP6P3* (Müller *et al*. 2008), *CYP6M2* (Stevenson *et al*. 2011), *CYP6Z1* (Nikou *et al*. 2003), *CYP325A3* (David *et al*. 2005; Awolola *et al*. 2009) and others (Djouaka *et al*. 2008; McLaughlin *et al*. 2008). The molecular basis of DDT resistance in *Drosophila* has been attributed to increased copy number and *cis*‐regulatory variants at the P450 *Cyp6g1* (Schmidt *et al*. 2010). Optimal insecticide resistance appears to involve the combination of multiple genes and mechanisms, including *kdr* (Corbel *et al*. 2007; Namountougou *et al*. 2012). For example, there is evidence that the combination of elevated P450 activity and *kdr* can confer a nonadditive increase in insecticide resistance (Hardstone *et al*. 2008). A recent report from the World Health Organization has stated that malaria vectors with both target‐site and metabolic resistance (e.g. *kdr* and P450) likely present the biggest threat to mosquito control efforts (WHO 2012).

We hypothesized that selection from increased ITN usage acted on multiple loci in *A. coluzzii* including those that have introgressed from *A. gambiae* in 2006 as well as on standing variation. To test this, we conducted a longitudinal study including whole‐genome sequencing and population‐scale genotyping of *A. gambiae* and *A. coluzzii* individuals collected both before and after the start of the 2006 ITN campaign in Selinkenyi, Mali. In addition, we conducted insecticide resistance bioassays to establish resistance phenotypes associated with the genotypes under study.

## Materials and methods

### Mosquito collections

Blood‐fed female mosquitoes were collected from inside human dwellings using mouth aspirators in Selinkenyi (11.700N, 8.2833W) and an adjacent (<25 km) village, Kela (11.8868N, 8.4474W), in Mali, during the rainy season (August–October). Mosquitoes were held until half‐gravid (60–70% digestion of bloodmeal), and the ovaries were removed and stored in Carnoy's solution (1 part glacial acetic acid and 3 parts 100% ethanol). The remaining carcass was stored in individual tubes containing 80% ethanol and transported to UC Davis for DNA extraction using the Qiagen Biosprint 96 system with Qiagen blood and tissue kits (Qiagen, Valencia, CA, USA). *Anopheles gambiae* and *Anopheles coluzzii* were distinguished from other *Anopheline* species using a diagnostic PCR developed by Scott *et al*. 1993 (Scott *et al*. 1993).

### Cytogenetic analysis

To estimate the frequency of the Bamako form of *A. gambaie* over time, polytene chromosomes were extracted from ovarian nurse cells using the protocol described by Hunt (Hunt 1973). Chromosome banding patterns were examined using an Olympus BX‐50 phase contrast microscope. The genotypes of five chromosome inversions – 2Rj, 2Rb, 2Rc, 2Rd and 2Ru – on the right arm of chromosome 2 (2R) were scored for individual mosquitoes. Individuals that were homozygous for 2R j, c and u inversions were identified as the Bamako form (Toure *et al*. 1998; Lee *et al*. 2013a; see supplemental information).

### Genotyping

To identify species and admixed individuals, we genotyped 458 mosquitoes from Selinkenyi, Mali, using the divergence island SNP (DIS) method described by Lee *et al*. (Lee *et al*. 2013b) with four additional SNPs at *CYP9K1* that distinguish three major haplotypes and two additional SNPs in the *para* gene that distinguish L1014F and L1014S *kdr* mutations. Species designation was determined based on fixed SNPs on the X chromosome (Favia *et al*. 1997, 2001; Fanello *et al*. 2002; Santolamazza *et al*. 2004, 2008). The informative SNPs for *CYP9K1* haplotypes were identified by visual inspection of paired‐end reads using the Integrated Genomics Viewer (IGV) (see Table S3 for assay design details and primer sequences). The Veterinary Genetics Laboratory at UC Davis conducted the Sequenom iPLEX SNP genotyping for this modified DIS method. *CYP9K1* haplotypes were determined using phase (version 2.1 and Stephens *et al*. 2001; Stephens & Donnelly 2003). DIS and *kdr* genotypes were plotted using matplotlib (Hunter 2007) following the colour scheme used in Lee *et al*. (2013b) and Norris *et al*. (2015). The Bamako and Savanna forms of *A. gambiae* were determined based on genotype data and by karyotyping (see Cytogenetic analysis).

### Genomic DNA library preparation and sequencing

Based on SNP genotype data, we selected 29 *A. coluzzii* individuals for genome sequencing: 12 pre‐2006 *A. coluzzii* and 17 post‐2006 *A. coluzzii*. In addition, we sequenced 7 *A. gambiae* individuals for a copy number analysis. Genomic DNA was quantified using a qubit 2.0 fluorometer (Life Technologies). DNA was cleaned and concentrated with Zymo Research DNA Clean and Concentrator kit. We used 25–50 ng of input DNA from individual mosquitoes for library construction. Genomic libraries were made with the Nextera DNA Sample Preparation Kit (Illumina) with TruSeq dual indexes (Illumina), modified to half volume. Libraries were size‐selected with Agencourt AMPure XP beads (Beckman Coulter). The concentration of finished libraries was quantified using a qubit 2.0 fluorometer. The expected library fragment size distribution was evaluated using a QIAxcel instrument (Qiagen) and Bioanalyzer 2100 (Agilent). Barcoded individual libraries were sequenced with the Illumina HiSeq2500 platform with paired‐end 100–150 bp reads at the QB3 Vincent J Coates Genomics Sequencing Laboratory at UC Berkeley (see Table S1 for raw sequence output per sample).

### Genome sequence analysis

We assessed the quality of our genome sequencing reads using the fastqc software (Andrews 2010). Adaptor sequences and poor quality sequence was trimmed from the raw reads using the trimmomatic software, version 0.30 (Bolger *et al*. 2014), with default options. Reads were aligned to the *A. gambiae* reference genome (AgamP4) using BWA‐mem (Li 2013). We used the MarkDuplicates module from Picard tools to remove PCR duplicates and the genome analysis tool kit (gatk) v1.7 to realign reads around indels (McKenna *et al*. 2010). The resulting sorted bam (Binary sequence Alignment/Map) files, which contain sequences for each read and its mapping position, were used for analysis.

*F*_ST_ between pre‐ and post‐2006 *A. coluzzii* was calculated in 50 kb windows with a 25‐kb step using the Weir and Cockerham estimator of *F*_ST_ (–weir‐pop‐fst) in vcftools (v0.1.11). We estimated Tajima's *D* using vcftools (–Tajima *D*) for both pre‐ and post‐2006 *A. coluzzii* and calculated the *standardized difference of D* (Δ*D*) with the following equation adopted from Bigham *et al*. (Bigham *et al*. 2010):

$$\Delta D=\left(\left(D_{\mathrm{iA}}-D_{\mathrm{iB}}\right)-u\left(D_{A}-D_{B}\right)\right)/SD\left(D_{A}-D_{B}\right)$$

where *D*_iA_ is Tajima's *D* for a given bin for pop A, *D*_iB_ is Tajima's *D* for a given bin for pop B, and *u* and *SD* are the mean Tajima's *D* and standard deviation for all bins from both populations. The step function was not available for Tajima's *D* with VCFtools, so a smaller window size (25 kb) was used. The data were plotted with Gaussian smoothing.

To elucidate copy number variation at the selected *cyp‐l* haplotype region, we analysed normalized sequencing coverage from whole‐genome sequencing data for *A. gambiae* (*N *= 7), pre‐2006 *A. coluzzii* (*N *= 12) and post‐2006 *A. coluzzii* (*N *= 17) individuals. To call individual duplicated regions, we used cnvnator (v0.3; Abyzov *et al*. 2011) with a bin size of 200 bp. We filtered for high‐quality calls using a *t*‐test *P*‐value threshold of 0.01, the size had to be >1 kb, and the default q0 filter was applied (calls with >50% reads with low mapping quality were ignored).

### Insecticide bioassays

Gravid *Anopheles* mosquitoes resting inside houses were collected using mouth aspirators in Selinkenyi, Mali, in August 2014 and individually housed in a glass vial or a microtube for oviposition. Mothers were saved in 80% ethanol after oviposition. 1st instar larvae were brought to UC Kearney. Three 2nd or 3rd instar larvae from each family were preserved in 80% ethanol for DNA extraction. We extracted DNA from the mother and three larvae from each family to genotype for species and hybrid status (DIS method), *kdr* and *CYP9K1*. Using these results, families with like genotypes were combined. In total, we generated 4 colonies with the following homozygous genotypes (species: *CYP9K1* haplotype: *kdr* status): (i) *A. coluzzii*:*cyp‐l*:*kdr*, (ii) *A. coluzzii*:*cyp‐l*:*wt*, (iii) *A. coluzzii*:*cyp‐ll*:*wt* and (iv) *A. gambiae*:*cyp‐lll*:*kdr*.

Permethrin and deltamethrin bioassays were performed on 6‐week‐old adult individuals that were first generation from the field and 1‐ to 3‐week‐old colony‐based individuals. Insecticide bottle bioassays were performed either on females or on a mix of adult males and females (see Supporting Materials) following the protocols of Brogdon & McAllister (1998). Briefly, 250 mL Wheaton bottles (Wheaton Industries, Millville, NJ, USA) were prepared by coating each with permethrin or deltamethrin dissolved in acetone at the WHO diagnostic dose (21.5 mg/mL and 12.5 mg/mL, respectively) or acetone alone (control). Bottles were left open for 1 h to evaporate residual acetone prior to bioassays. A group of 6–20 individual mosquitoes were introduced into each bottle, and the number of individuals that were nonresponsive upon disturbing the bottle (knocked down) and rotating it horizontally 360 degrees was recorded at five‐minute intervals. The time when 50% and 90% of the mosquitoes were knocked down (KD_50_ and KD_90_, respectively) within a given bottle was determined using a best fit curve. The plotted KD_50_ and KD_90_ values are the mean and standard error between replicates. Significant differences between knock‐down times was calculated using a 2‐tailed *t*‐test.

## Results

### Temporal dynamics of species composition

**Figure 1:**
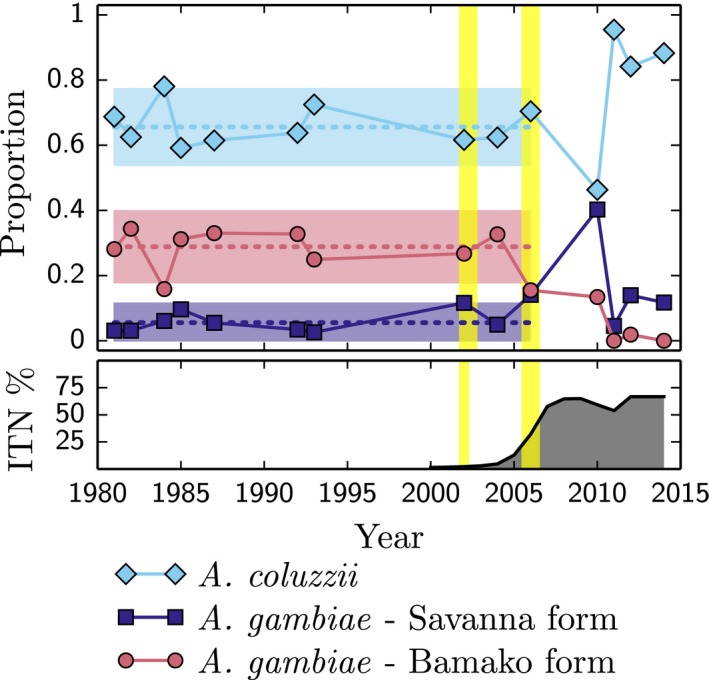
Temporal Dynamics of Species Composition at Selinkenyi, Mali. Shown is the relative abundance of the Savanna and Bamako Forms of *Anopheles gambiae* and *Anopheles coluzzii* collected from the town of Selinkenyi, Mali, between the years 1980 through 2014. Data prior to 1991 were taken from Toure *et al*. 1998;. Data from 1991 through 2010 were collected by us and reported in Lee *et al*. 2013a,b. Data since 2010 are new. The yellow vertical lines mark when F1 hybrids were observed. The bottom graph displays the estimated proportion of the population sleeping under insecticide‐treated bed nets (ITN). ITN usage data was taken from WHO (2013).

**Figure 2:**
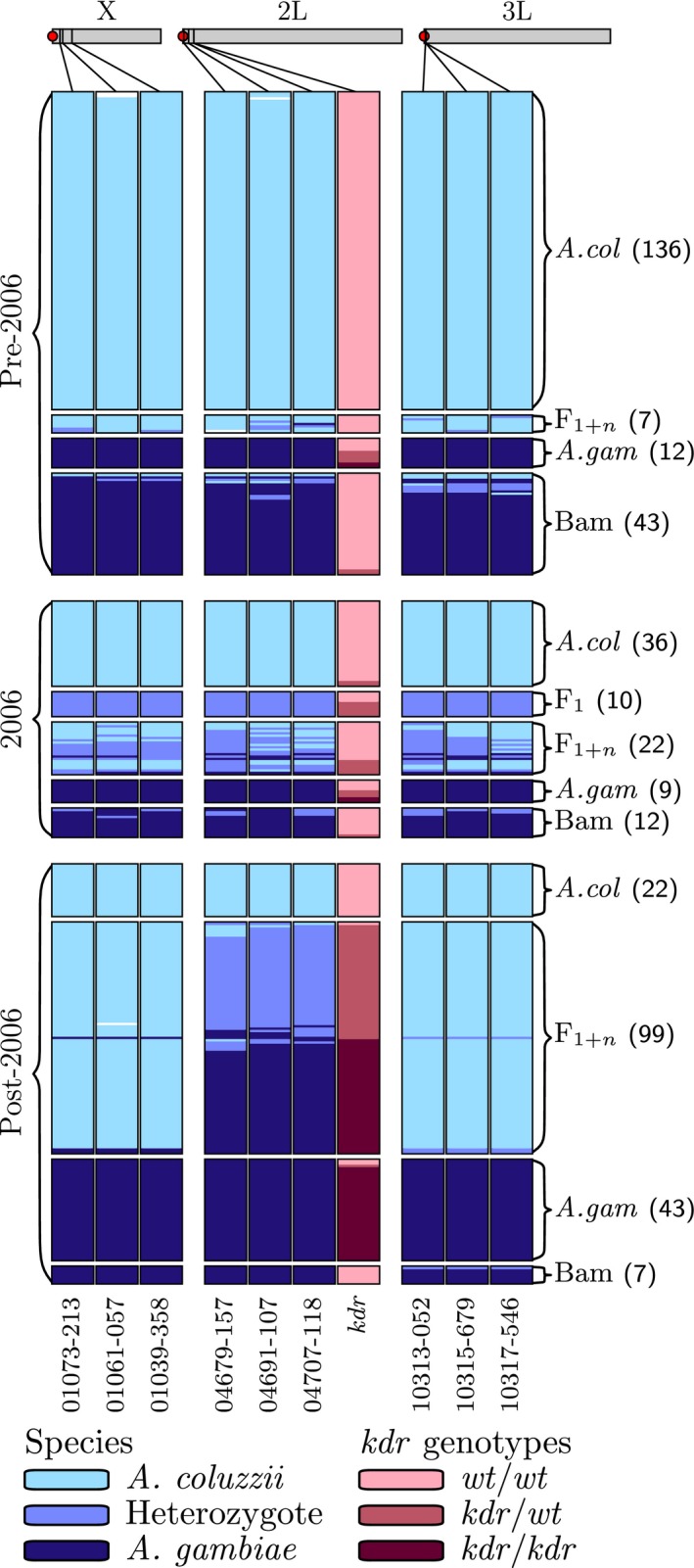
A Longitudinal Survey of Divergence Island SNP (DIS) and *kdr* Genotype Frequencies at Selinkenyi, Mali. Data are organized into three time periods: Top panel – pre‐2006 (includes data for 2002 and 2004); centre panel – 2006; bottom panel – post‐2006 (2010 and 2012). Individual SNPs are organized by chromosome location: X chromosome (*N *= 3), chromosome 2L (*N *= 4) and chromosome 3L (*N *= 3). Individual SNP identifiers are provided at the bottom of each column. Details for each DIS can be found in Lee *et al*. (2013a) and for the *kdr* SNP in Norris *et al*. (2015). Light blue = homozygous for *Anopheles coluzzii* DIS, medium blue = DIS heterozygote and dark blue = homozygous for *Anopheles gambiae* DIS. Dark red = homozygous *kdr* resistant (*kdr*), medium red = *kdr* heterozygote and pink = *kdr* susceptible (*wt*) homozygote. The chromosomal forms of *A. gambiae* are represented as follows: Bamako = Bam and Savanna = *A. gam*. F_1_ = first‐generation hybrid and F_1+*n*_ **= backcross genotypes. Sample sizes for each genotype are given in parentheses.

In 2005, the President's Malaria Initiative initiated a major ITN campaign in Mali (Flaxman *et al*. 2010; WHO 2013). To explore the potential relationship between this anthropogenic selection pressure on the relative fitness of *Anopheles coluzzii*,* Anopheles gambiae* and Ag‐Bamako, we plotted their relative abundance at our study site (Selinkenyi) based on adult mosquito collections starting in 1980 through 2014 (Fig. 1). These data were gathered from Touré *et al*. (Toure *et al*. 1998) and our own collections, some of which have been published (Lee *et al*. 2013a,b). For our analysis, we used only wet season collections (May–October). During the 25 years prior to 2005, the frequencies of *A. gambiae*, Ag‐Bamako and *A. coluzzii* in the population were remarkably stable, representing approximately 10%, 25% and 65%, respectively, at Selinkenyi (Figs 1 and 2). In 2006, we observed a punctuated burst of *A. coluzzii* x *A. gambiae* hybrids, including 16 F_1_s and 9 recombinants, *N *= 124 (Fig. 2). By 2010, early‐stage hybrid genotypes were no longer detected, but *A. coluzzii* individuals with *kdr* and the physically linked 2L island from *A. gambiae* were common (Fig. 2). The *A. coluzzii* population increased in relative frequency from approximately 65% pre‐2006 to 88% in 2014 (*N *= 179), likely due to the increased representation of the *kdr*‐introgressed *A. coluzzii* genotype (80%; *N *= 155). This gain in relative abundance of *A. coluzzii* is proportional to the decline of Ag‐Bamako from 25% in pre‐2006 to 0% in 2014 (*N *= 179, Fig. 1).

### Sequence differentiation between pre‐ and post‐2006 *Anopheles coluzzii*

**Figure 3:**
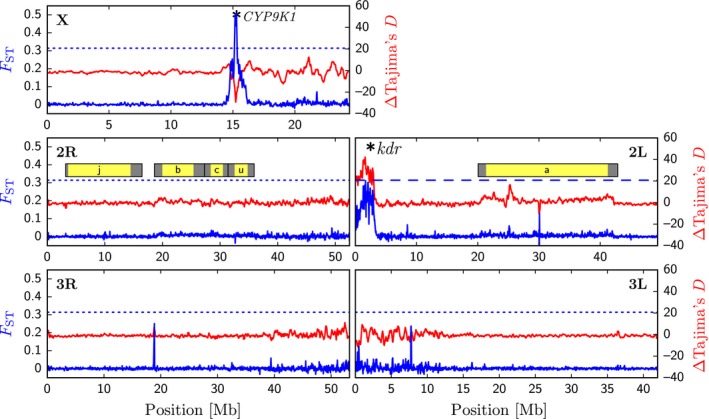
Sequence divergence between pre‐ and post‐2006 *Anopheles coluzzii*. Panels represent each of the three chromosomes (X, 2 and 3), as indicated by labels on the top outside corner of each box. *F*_*ST*_ were calculated in 50 kb windows with 25 kb steps comparing 12 ‘pure’ (pre‐2006) *A. coluzzii* and 17 *kdr Anopheles coluzzii* (post‐2006). *F*_*ST*_ is displayed in blue (with Gaussian smoothing). The 99.9% threshold for *F*_*ST*_ is indicated with a dashed line. The standardized difference of *D* (∆*D*) is a relative Tajima's *D* statistic (Bigham *et al*. 2010) shown in red. Negative ∆*D* values indicate fewer haplotypes than expected in post‐2006 samples vs. pre‐2006. The positions of common inversions are shown for reference and indicated in yellow with breakpoints highlighted in grey. Genes of interest are labelled with a star.

The *kdr*‐introgressed *A. coluzzii* genotype first appeared in 2006 in Selinkenyi. Since then, it has outcompeted *wt A. coluzzii*, reaching 97% in 2014 (*N *= 159). To elucidate additional introgressed regions (other than 2L) and/or selection on standing variation elsewhere in the *A. coluzzii* genome, we sequenced the genomes of 12 pre‐2006 *A. coluzzii* (*kdr* freq. = 0) and 17 post‐2006 *A. coluzzii* (*kdr* freq. = 0.56). All genomic libraries for this study were prepared from single, field‐collected adult females and were sequenced at a median depth of 14× (Table S1, Supporting information). To identify major differentiated regions between pre‐2006 and post‐2006 *A. coluzzii* genomes, we calculated *F*_ST_ and a relative Tajima's *D* statistic [∆*D*, standardized difference of *D* (Fig. 3; see Methods)]. In brief, negative ∆*D* values may be indicative of a selective sweep after 2006, while positive ∆*D* values could be due to an enrichment of heterozygotes (e.g. due to balancing selection) after 2006. This analysis revealed two prominent *F*_ST_ peaks, including the expected *kdr* locus within the speciation island on chromosome 2L (Fig. 3). Interestingly, we observed positive ∆*D* values at this pericentromeric 2L region.

The second differentiated region had high *F*_ST_ and negative ∆*D* values that were centred at approximately 15.24 Mb on the X chromosome. To approximately estimate the size of the region under selection in post‐2006 *A. coluzzii*, we examined a zoomed‐in region from 14.6 Mb to 16 Mb with *F*_ST_ at higher resolution (5 kb windows and 1 kb steps). Peak *F*_ST_ values (>95%ile) span a 156‐kb region (Fig. S1, Supporting information) including 4 genes: an uncharacterized gene (AGAP012997), a P450 gene (*CYP9K1*), a terminal gap gene (*Tailless*) and a cuticular protein (*CPR125*). We detected 30 nonsynonymous mutations among these genes. However, only one (I221T; rs5558865), located in AGAP012997, was associated with the selective sweep (Table S2, Supporting information). This gene has four known paralogs (AGAP013173, AGAP000816, AGAP000817 AGAP013424) located nearby (within 200 kb), but a well‐characterized orthologue was not identified. Although we can rule out nonsynonymous mutations in the remaining three genes associated with the selective sweep, it is possible that adaptive regulatory variation at these genes is the target of selection. Thus, *CYP9K1, Tailless* and *CPR125* remain important candidate genes under selection.

Visual inspection of long paired‐end reads using Integrative Genomics Viewer (IGV) revealed haplotype‐specific SNPs in the transcription start sites, intron and 3′UTR of *CYP9K1* and *Tailless*. To estimate the frequency of the highly selected haplotype in the populations, we used a custom Sequenom iPLEX genotyping assay to sequence 6 SNPs that span the *CYP9K1* 3′UTR and the *Tailless* gene, including two SNPs in the *CYP9K1* 5′ transcription start site (−47 bp, −100 bp). In parallel, we genotyped *kdr* and species‐specific SNPs in the speciation islands on each chromosome (Supplemental Data). Using this multiplex genotyping data, we identified three common haplotypes at the *CYP9K1* region (see methods) and estimated haplotype frequencies by species and collection year. Because *CYP9K1* is a primary candidate gene, we refer to the haplotypes as *cyp‐l*,* cyp‐ll* and *cyp‐lll* (Table 1). The genotype at synonymous *Tailless* SNPs were perfectly correlated with *CYP9K1* genotypes (*r* ^2^ = 1; *N *= 27 *A. coluzzii* and *N *= 21 *A. gambiae* from 2010), indicating that this highly selected haplotype spans at least *CYP9K1* and *Tailless*, but appears to commonly span approximately 156 kb in *A. coluzzii* collected in 2012 (Fig. S1, Supporting information).

**Table 1:** Frequency of *CYP9K1* haplotypes in pre‐ and post‐2006 individuals. To rule out the possibility of introgression of the *cyp‐l* haplotype from *Anopheles gambiae*, we genotyped 5 informative SNPs that span *CYP9K1* and *Tailless* (+9.4 kb) to assess the frequency of the *cyp‐l* haplotype in *Anopheles coluzzii* and *A. gambiae* pre‐ and post‐2006. Note that *cyp‐l* was common in *A. coluzzii* (4–23%) and not detected in *A. gambiae* prior to the 2006 hybridization event. Also, *cyp‐ll* was more common that *cyp‐l* pre‐2006

	Frequency of *CYP9K1* haplotypes, %							
	*A. coluzzii*				*A. gambiae*			
	2002	2004	2010	2014	2002	2004	2010	2014
*cyp‐l*	4	23	85	99	0	0	0	9
*cyp‐ll*	46	31	4	<1	0	0	0	0
*cyp‐lll*	48	39	12	0	100	97	100	87
*cyp‐other*	2	7	0	<1	0	3	0	4
*N*	26	35	26	158	13	18	21	21

### Distinguishing Introgression from selection on standing variation

To determine whether the differentiated locus on the X chromosome was due to introgression, like the previously described introgression of *kdr* and 2L island on chromosome 2 (Norris *et al*. 2015), we assessed the frequency of the *cyp* haplotypes and *kdr* in the *A. coluzzii* and *A. gambiae* specimens collected from 2002 to 2014 using the iPLEX SNP genotyping assay described above (Table 1). In total, we genotyped 87 *A. coluzzii* and 52 *A. gambiae* individuals. The *cyp‐l* haplotype has increased in frequency in *A. coluzzii* from 4% in 2002 to 99% in 2014. The *cyp‐lll* haplotype was the predominant haplotype (>89%) in *A. gambiae* in all years. The *cyp‐l* haplotype was not detected in *A. gambiae* prior to 2014 (*N *= 52).

### Insecticide resistance bioassays

**Figure 4:**
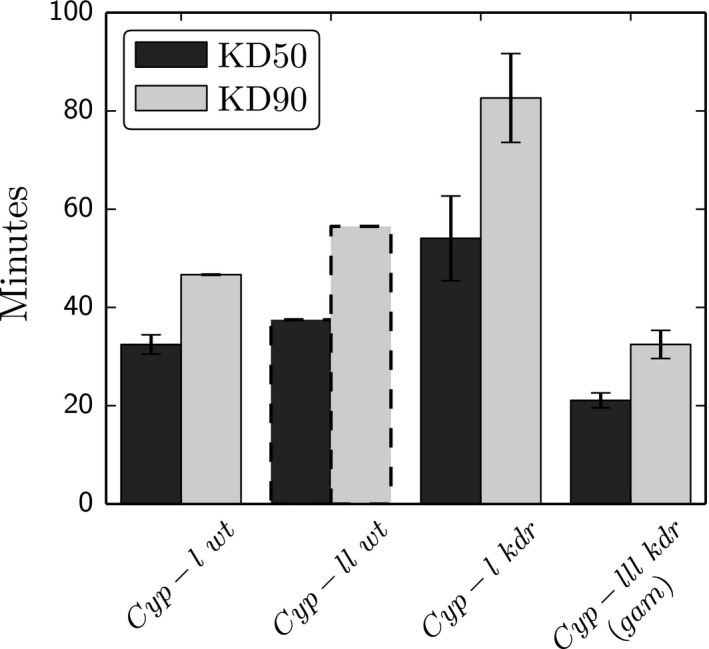
Insecticide resistance bioassay results. KD_50_ is the estimated time (minutes) when 50% of the mosquitoes in a given insecticide (permethrin) coated bottle were nonresponsive (knocked down) after holding the bottle horizontally and rotating the bottle 360 degrees. These mixed gender assays were first‐generation offspring from field‐collected females. Shown are mean KD_50_ (black) and KD_90_ (light grey) for specific genotypes with standard error. The *CYP9K1* haplotype (*cyp‐l, ll or lll*) and *kdr* status are noted below each bar. Note the small sample size of *cyp‐ll:wt* (dashed bar; one replicate of *N *= 7).

To determine if elevated insecticide resistance is associated with the increase in the relative frequency of *kdr*:*cyp‐I A. coluzzii* individuals, we performed insecticide resistance bioassays (see methods) on 4 recently derived mosquito colonies representing the following homozygous genotypes (species: *CYP9K1* haplotype: *kdr* status): (i) *A. coluzzii:cyp‐l:kdr*, (ii) *A. coluzzii:cyp‐l:wt,* (iii) *A. coluzzii:cyp‐ll:wt* and (iv) *A. gambiae:cyp‐lll:kdr*. Some genotypes (e.g. *A. gambiae:cyp‐lll:wt* and *A. coluzzii:cyp‐ll:kdr*) were not evaluated because they were not present in our collection. The *cyp‐l:kdr A. coluzzii* individuals were significantly more resistant than *cyp‐l:wt A. coluzzii* (*t*‐test; *P *=* *0.01) and *cyp‐lll:kdr A. gambiae* (*t*‐test; *P *<* *0.0001) at both KD_50_ and KD_90_ (Fig. 4). The *cyp‐l:wt A. coluzzii* (*N *= 40) genotype is trending towards slightly less resistance than *cyp‐ll:wt A. coluzzii* (*N *= 7), but more replicates are needed to assess significance (dashed bar; Fig. 4). For external reference, *cyp‐l:kdr A. coluzzii* was also highly resistant compared the *cyp‐ll:wt A. coluzzii* MOPTI colony (MRA‐763; 0% vs. 100% KD at 30 min; *N *= 20, 19). Resistance was not limited to permethrin as *cyp‐l:kdr A. coluzzii* were also more resistant than *cyp‐lll:kdr A. gambiae* under deltamethrin exposure (12.5 μg/bottle; 0% vs. 85% KD at 30 min; *N *= 20, 20). The bioassays in Fig. 4 were performed on a mix of male and females due to sample size limitations with some genotypes. As there may be gender‐specific differences in KD times, more bioassays are needed to accurately quantify the relative contributions of the three *CYP9K1* haplotypes to insecticide resistance with or without *kdr*.

### Analysis of copy number variation

To test for copy number variation specifically at the *CYP9K1* region in (i) *A. gambiae*, (ii) pre‐2006 *A. coluzzii* and (iii) post‐2006 *A. coluzzii*, we examined relative sequencing depth across the genome in 200 bp bins for each individual (see Methods). Using this approach, we did not detect multiple copies at the *CYP9K1* region in pre‐2006 *A. coluzzii* or post‐2006 *A. coluzzii*. However, a 21‐kb region, including *CYP9K1* in *A. gambiae,* had normalized read depth of 2.5 (*t*‐test *P*‐value *<*0.0001, *N *= 7), indicating a duplication at this region in *A. gambiae* (Table S2, Supporting information). As *A. gambiae* is nearly fixed for *cyp‐lll*, individual paralogs within its genome are likely not confounding our genotyping results.

## Discussion

### Adaptive introgression of *kdr*

Using fixed SNP markers within the pericentromeric ‘speciation islands’ on X, 2L and 3L, a longitudinal study identified punctuated bursts of F1 hybrids between *Anopheles coluzzii* and *Anopheles gambiae* in Selenkenyi, Mali (Lee *et al*. 2013b). In 2006, local ITN use dramatically increased coincident with a particularly large burst of hybrids (Fig. 1). By 2010, linkage disequilibrium (LD) between the X and 3L islands was re‐established, but the entire 2L island was lost, replaced by the *A. gambiae* island containing *kdr* (Fig. 2). It has been hypothesized that increased ITN usage altered the fitness landscape, resulting in a relative fitness increase of normally unfit hybrid genotypes (Norris *et al*. 2015). The subsequent increase in relative abundance of *A. coluzzii* (Fig. 1) and elevated insecticide resistance of *kdr A. coluzzii* individuals (Fig. 4) indicate that this introgression event is adaptive. The burst of typically unfit hybrids detected in 2006 (Lee *et al*. 2013b) likely produced myriad mixed genotypes upon which strong selection could act. Ultimately, only *A. coluzzii* (based on fixed X chromosome markers) that had the 2L introgression (with *kdr*) from *A. gambaie* and the *A. coluzzii* 3L island persisted in the population, resulting in the re‐establishment of LD between the X and 3L islands. This is an important observation because the maintenance of LD between unlinked loci in the face of gene flow is a critical requirement for divergence with gene flow (i.e. the speciation islands model).

### Introgression vs. selection on standing variation

For an initial assessment of sequence differentiation between pre‐ and post‐2006 *A. coluzzii,* we analysed *F*_ST_ in sliding windows (see Methods). This approach revealed a prominent differentiated region at the expected *kdr* locus and linked 2L island and a second region at approximately 15.24 Mb on the X chromosome, centred at *CYP9K1*. To reveal signatures of recent selection in the *A. coluzzii* genome, we used ∆*D,* a relative Tajima's *D* statistic (see Methods). The relationship between *F*_ST_ and ∆*D* was different between the X and 2L regions. At the known introgression on 2L, there was a positive relationship, with elevated *F*_ST_ and ∆*D*. The positive Tajima's *D* trend in post‐2006 *A. coluzzii* is likely due to the increased representation of individuals that were heterozygous for the 3 Mb island (10/17 were heterozygous for *kdr*). In addition, model‐based estimates predict that the acquisition of a selected allele from a differentiated population (like *kdr* from *A. gambiae*) can result in elevated Tajima's *D* values at sequences linked to the selected allele (Santiago & Caballero 2005).

On the X, ∆*D* and *F*_ST_ were negatively associated. The dip in ∆*D* in this case was due, at least in part, to the near fixation of a haplotype (*cyp‐l* hereafter) in post‐2006 *A. coluzzii*, which spans at least four genes including AGAP013173, *CYP9K1*,* Tailless* and *CPR125*. The *cyp‐l* haplotype was present in *A. coluzzii* at 23% in 2004 (*N *= 35) and was not detected in *A. gambiae* prior to 2014 (*N *= 52), indicating that selection acted upon standing variation within *A. coluzzii* (Table 1). Continued population sampling is needed to assess whether the *cyp‐l A. gambiae* genotype detected in 2014 will be selected for in the population and warrant further study. We hypothesize that selection has acted upon *cis*‐regulatory variation at *CYP9K1* because (i) *CYP9K1* has been shown to be upregulated in resistant anopheline mosquitoes (Tene *et al*. 2013; Mulamba *et al*. 2014), (ii) the dramatic increase in the *cyp‐l* haplotype was coincident with the increase in ITN usage in 2006, and (iii) bioassay results confirm that *kdr* alone cannot account for the level of resistance observed for *kdr:cyp‐l A. coluzzii* genotype. That said, the nonsynonymous mutation in AGAP013173 and regulatory variation at *Tailless* or *CPR125* should also be considered good candidates for selection.

### Evidence for synergistic epistasis

In 2004, the *A. coluzzii* population was *kdr* susceptible (*N *= 61) and variation at the *CYP9K1* locus was composed of three major haplotypes: *cyp‐l* (23%), *cyp‐ll* (31%) and *cyp‐lll* (39%) (*N *= 35; Table 1). After 2006, the *cyp‐I:kdr* genotype appears to be approaching fixation in *A. coluzzii* and the *cyp‐II* genotype with the *kdr* introgression (*cyp‐ll:kdr*) was never observed in this species. This is despite the fact that the frequency of *cyp‐ll* (31%) was higher than *cyp‐l* (23%) in pre‐2006 *A. coluzzii*. Insecticide resistance bioassays indicate that the *cyp‐l A. coluzzii* with the *kdr* introgression is more resistant than any other genotype tested, including *A. gambiae* (*cyp‐lll*) with *kdr* (Fig. 4). This result supports the hypothesis that selection for insecticide resistance is contributing to the observed changes in the modern *A. coluzzii* genome and altering long‐standing species dynamics in Selenkenyi, Mali (Fig. 1). This is also evidence that resistance in *A. coluzzii* is more complex than *kdr* alone as *A. gambiae* has *kdr*, but is less resistant and has not increased in relative frequency as of 2014 estimates (Fig. 1). Bioassay results comparing *kdr*‐susceptible colonies (or families) with homozygous *cyp‐I* or *cyp‐II* genotypes indicate that *cyp‐I* may not offer increased resistance compared to *cyp‐II* in the absence of *kdr* (Fig. 4). So why did the *cyp‐l* haplotype dramatically increase in relative frequency? One hypothesis is that the exclusive selection for *cyp‐l* in the presence of *kdr* is due to an allele‐specific interaction between *kdr* and *cyp‐I* resulting in a nonadditive increase in resistance. Synergistic epistasis between P450 genes and *kdr* has been described previously in *Culex* mosquitoes (Hardstone *et al*. 2008), further supporting the possibility that *CYP9K1* is the target of selection within the *cyp‐l* haplotype. Another hypothesis is that alleles in the *cyp‐l* haplotype were selected for because they were the most compatible with the *A. gambaie* alleles in the 2L island in nature. We also cannot rule out the possibility that selection for the *cyp‐l* haplotype could have been independent of the 2L introgression and *kdr* (no epistasis).

Understanding the genetic basis for the potential interaction between *kdr* and *CYP9K1* is important because the World Health Organization recently suggested that insecticide resistance via the combination of *kdr* and elevated P450 activity represents the biggest threat to vector control for malaria in Africa (WHO 2012). The latest ITNs add piperonyl‐butoxide (PBO), a general P450 inhibitor, to better combat complex insecticide resistance (e.g. PermaNet^®^ 3.0). Functional verification of the nonsynonymous mutation in AGAP013173 as well as regulatory variation in the four candidate genes within the *cyp‐l* haplotype is an important next step beyond this study to confirm that selection is acting on *CYP9K1* and/or other linked loci. For example, allele‐specific gene expression assays between pairwise hybrids of the *cyp‐l*,* cyp‐ll* and *cyp‐lll* haplotypes would be ideal to estimate the effects of the 5′ proximal SNPs on gene expression.

We described evidence for elevated copy number at the *CYP9K1* region exclusively in *A. gambiae*. Variation in P450 copy number appears common between species (Good *et al*. 2014). A population‐scale analysis of copy number variation at the *CYP9K1* region in *A. gambiae* (e.g. via qPCR) would reveal whether the detected duplication is fixed in the population and whether higher copy genotypes exist. It would be interesting if the lack of *cyp‐l* in *A. gambiae* is partially compensated for by an *A. gambiae* ‐specific increase in copy number, but bioassay data indicate that *cyp‐lll:kdr A. gambiae* is significantly less resistant to insecticide than *cyp‐l:kdr A. coluzzii* (Fig. 4). Thus, this copy number variation may be unrelated to insecticide resistance. Metabolic studies would also be very informative as *CYP9K1* is closely related to, but not among the several P450 genes that have been proven to metabolize pyrethroid insecticides in vitro (Müller *et al*. 2008; Stevenson *et al*. 2011).

### Intraspecific mating fidelity and evolutionary responsiveness

*Anopheles gambiae*, Ag‐Bamako and *A. coluzzii* have fairly consistently represented 10%, 25% and 65%, respectively, of the mosquito population at Selinkenyi, based on collection data from 1980 to 2006 (Fig. 1). *Ag‐Bamako* appears to be part of an adaptive radiation in the *A. gambiae* species complex via its adaptation in the larval stage to riverine rock pools (Toure *et al*. 1998; Manoukis *et al*. 2008). The time since divergence between *Ag‐Bamako* and *A. gambiae* is thought to be much more recent than that between *A. gambiae* and *A. coluzzii* (Taylor *et al*. 2001; Slotman *et al*. 2006). Ag‐Bamako is identified primarily by the presence of the j inversion on chromosome 2R and is *A. gambiae* ‐like on the X chromosome. Based on karyotype and genotype data, we demonstrate that the longstanding species dynamics between these three populations has changed following the start of the major ITN campaign in 2006 (Fig. 1). In 2002 and 2004, *A. coluzzii* and Ag‐Bamako populations were *kdr* susceptible (*N *= 142 and 43, respectively), whereas the *kdr* frequency in *A. gambiae* was approximately 50% (*N *= 12). In post‐2006 samples, leaky reproductive barriers between *A. gambiae* and *A. coluzzii* and strong selection resulted in the stable introgression of *kdr* into *A. coluzzii*. The *kdr A. coluzzii* genotype is now significantly more resistant to insecticides (Fig. 4) and has increased in relative frequency in the population from 65% to 97% in 2014 (*N *= 179; Fig. 1). We suggest that the brief increase in the relative frequency of *A. gambiae* in the population after 2006 may be due to the presence of relatively unfit early‐stage mixed *A. coluzzii* genotypes. Unlike *A. gambiae* and *A. coluzzii*, reproductive isolation appears to be nearly complete between Ag‐Bamako and the other two taxa in Mali (Toure *et al*. 1998; Powell *et al*. 1999; Manoukis *et al*. 2008). The high mating fidelity in Ag‐Bamako appears to have prevented the acquisition of *kdr* from either *A. coluzzii* or *A. gambiae* (*N *= 7; Fig. 2), which may be responsible for its steady decline towards local extinction in Selinkenyi, Mali. Thus, unstable reproductive barriers resulting in adaptive introgression of *kdr* appear to have enabled *A. coluzzii* to adapt to a rapid environmental change (i.e. increased ITN use) and even out‐compete Ag‐Bamako, a population associated with the donor species, *A. gambiae*.

## Conclusion

Our results indicate that extant *kdr A. coluzzii* populations in Mali are highly resistant to insecticides (both permethrin and deltamethrin) and have increased in relative frequency in the presence of increased ITN usage. We hypothesize that this elevated insecticide resistance is due to interactions between the introgressed *kdr* allele and allele/s within the *cyp‐l* haplotype on the X chromosome*,* which was already present in the *A. coluzzii* population. Thus, surveys of insecticide resistance in malaria vectors may benefit from assessing the population frequencies of both *kdr* and the *cyp‐l* haplotype. That said, it is also possible that increased ITN use drove the introgression of the *A. gambiae* 2L island containing *kdr*, and then, variable genetic incompatibilities between *A. gambiae* alleles in the 2L island and alleles in the *CYP9K1* region resulted in fixation of the most amenable haplotype (*cyp‐l*). We also cannot rule out selection from other environmental changes, for example climate change or new pathogens. The remarkable adaptive radiation of the *A. gambiae* mosquito complex and leaky reproductive barriers between species may underlie their resilience to rapid environmental changes and ultimately their persistence through prehistory.

B.J.M., Y.L. and G.C.L. designed research; B.J.M., Y.L. and T.C.C. performed the data analysis; G.C.L., A.F. and A.J.C. carried out field collections; A.F. and A.J.C. performed the cytogenetic analysis; B.J.M. and K.B. performed the bioassays; B.J.M., Y.L., A.J.C., L.C.N. and G.C.L. wrote the paper.

## Data accessibility

Illumina sequencing data were deposited in SRA at NCBI under accession number SRP063464. Variant data has been deposited in Dryad (http://dx.doi.org/10.5061/dryad.f3dn2). Other data associated with this study are available in supporting information and in the *PopI* open online database (https://popi.ucdavis.edu/PopulationData/OpenProjects/AgKDR/).

## Supporting information


**Table S1** Genome sequencing reads per sample.
**Table S2** Candidate nonsynonymous mutations.
**Table S3** Sequenome iPLEX primer design.
**Fig. S1** Estimating the size of the cyp‐l haplotype.
**Data S1** Haplotype estimates from genotype data at the CYP9K1 region.
**Data S2** Bioassay data.
**Data S3** Copy number analysis using cnvnator (v0.3).
